# Knowledge, attitudes and practice of clinicians managing chronic pain in a tertiary care facility

**DOI:** 10.4102/sajp.v78i1.1597

**Published:** 2022-01-31

**Authors:** Solomon Rop, Joseph M. Matheri, Nassib Tawa

**Affiliations:** 1 Department of Rehabilitation Science, College of Health Sciences, Jomo Kenyatta University of Agriculture and Technology, Nairobi, Kenya; 2 Division of Physiotherapy, Faculty of Medicine and Health Sciences, Stellenbosch University, Cape Town, South Africa

**Keywords:** chronic pain, knowledge, attitudes and practices, pain, pain management, assessment, clinician

## Abstract

**Background:**

Chronic pain is a common clinical symptom and has a high socio-economic and health burden on patients, clinicians and the healthcare sector. Globally, clinicians continue to exhibit limited knowledge, negative attitudes and misconceptions about chronic pain, raising public health concerns.

**Objective:**

Our study aimed to determine the level of knowledge, attitudes and practices of clinicians towards assessment and management of patients with chronic pain in a tertiary hospital.

**Methods:**

This cross-sectional study at a tertiary care hospital in Kenya adopted a census method to recruit participants (*n* = 240). A questionnaire containing 77 items derived from the Revised Pain Knowledge and Attitudes Questionnaire (RPKAQ) and the Knowledge and Attitudes Survey regarding Pain (KASRP) was used to collect data. The questionnaire had three parts. Part A had six items to determine information on demographic characteristics. Part B and C had 54 and 23 items, respectively, that determined information on knowledge, attitudes and practice of clinicians managing chronic pain. Data were analysed using SPSS Version 24.

**Results:**

Response rate was 83.3% (*n* = 153). Only 9% (*n* = 14) of the respondents were believed to have adequate knowledge and positive attitudes; 62% (*n* = 95) used best practice for cognitive/behavioural management of chronic pain. Few (9% *n* = 14) used best practice in the assessment and measurement of chronic pain.

**Conclusion:**

There is inadequate knowledge, attitudes and practices amongst clinicians regarding assessment and management of chronic pain. Future research is needed in a wider population to compare these results.

**Clinical implication:**

It may motivate clinicians to improve their level of knowledge, attitudes and practices for pain management, hence improving poor chronic pain outcome.

## Background

Despite the recent advances in understanding the algorithm of diagnosing and treating clinical pain, many clinicians globally continue to exhibit limited knowledge, negative attitudes and misconceptions about chronic pain, raising public health concerns (Al-Quliti & Alamri 2015; Clenzos, Naidoo & Parker 2016; Magalhães et al. 2012; Nuseir, Kassab & Almomani 2016; Ung et al. 2016). Chronic pain is a common clinical symptom reported by patients in referral facilities and has a significantly high socio-economic and health burden on patients, clinicians and the healthcare sector (Goldberg & McGee 2011). Pain is defined as ‘an unpleasant sensory and emotional experience associated with actual or potential damage of tissue’ (International Association for the Study of Pain 2015). Regarding the relationship between chronic pain and tissue damage, ‘the pain does not provide a measure of state of the tissue as it is modulated by many factors, which include somatic, psychological and social factors’. Therefore, ‘the relationship between pain and tissue becomes less predictable as pain persists’ (Moseley 2008). According to the International Association for the Study of Pain, Treede et al. (2018) and Raja et al. (2020) views are that the ‘sensory and emotional aspect of chronic pain are thought to be without real biological cause and persist beyond the normal time for tissue healing’.

Patients suffering from chronic pain often report increasing levels of pain, activity limitation, participation restrictions and poor quality of life, which comes with high medical costs as a result of prolonged utilisation of healthcare services (Salazar, Mico & Failde 2016). In practice, the outcomes of patients with chronic pain are often poor because of inaccuracy amongst clinicians in choosing and administering appropriate treatment (Kheshti et al. 2016). According to Breivik et al. (2006) and Nuseir et al. (2016) inaccurate prescription for patients with chronic pain may be a function of misclassification or misdiagnosis, which leads to inappropriate treatment and undesirable outcomes and increased health and social costs. Whereas the goal when managing people suffering from chronic pain is to relieve pain and improve function, a bio-psychosocial approach is currently preferred for improved outcome especially when provided by a multidisciplinary team (Bevers, Watts & Gatchel 2016; Ernstzen, Louw & Hillier 2017; Espejo-Tort et al. 2011; Moseley 2008). However, research evidence has shown that most clinicians utilise a biomedical approach, which is regarded as a retrogressive practice that is often associated with poor outcomes, reduced quality of life, increasing disability and the high cost of healthcare (Bevers et al. 2016; Espejo-Tort et al. 2011). According to Gustafsson and Borglin, (2013) improving clinicians’ level of knowledge positively influences their attitudes and practice for effective management of patients with chronic pain. However, most of the studies with these outcomes were conducted in developed countries such as in the United Kingdom (Ryan et al. 2010), United States of America (Duke et al. 2013) and Asia (Al-Quliti & Alamri 2015). In the African region, information regarding knowledge, attitudes and practices of healthcare professionals about assessment and management of patients with chronic pain is scanty. To address this gap, our study aimed to determine the level of knowledge, attitudes and clinical practices on assessment and management of chronic pain amongst clinicians in a tertiary care facility in Kenya. Our study’s findings form an essential baseline for further research and inform future review of healthcare policy, capacity development programmes and practice.

## Method

Our cross-sectional survey was conducted at Tenwek Hospital in Kenya from January 2020 to May 2020. We targeted the 240 different healthcare professionals who are directly involved in caring for patients within the hospital (as illustrated in Table 1).

**TABLE 1:** Study population (*n* = 240).

Professional	Number
Medical officers, residents and specialists	50
Nurses	124
Clinical officers (physicians’ assistants)	40
Anaesthetists	15
Physiotherapists	4
Oncology team	7

**Total**	**240**

We adopted a census method of sampling because of the small study population (*n* = 240). Although this method is expensive and time-consuming it has no sampling error commonly seen in probability sampling methods and gives precise results. In addition, the census method is best used for heterogeneous populations (Ajay & Micah 2014). Clinicians were included if they were directly caring for patients with chronic pain and were willing to participate and voluntarily gave signed consent.

### Data collection tool

The revised Pain Knowledge and Attitude Questionnaire (RPKAQ) and a section adopted from the Knowledge and Attitudes Survey regarding Pain (KASRP) questionnaire, which mainly focuses on pharmacological management of pain was used to collect data. Both the RPKAQ and KASRP questionnaires have undergone cognitive testing and validation in different settings and cultures and have been found to be reliable (Cronbach’s alpha of 0.65 and 0.80, respectively); (Clenzos et al. 2016; Ferrell & McCaffery 2012). The RPKAQ was designed to measure wide knowledge, attitudes and practices appropriate for clinicians, which includes the physiological basis of pain, psychological factors of pain perception, the developmental changes, the assessment and measurements of pain and cognitive and behavioural methods of pain management and pharmacological management of chronic pain. Each item was scored and the total ranges from 0% to 100%. A score of 75% and above represents acceptability and it means the participant has adequate knowledge, attitudes and practices whilst those who score below 75% are considered to have inadequate knowledge, negative attitudes and poor practices towards assessment and management of patients with chronic pain.

### Procedure for data collection

Prior to data collection, the first author sought permission to access clinicians from the hospital’s human resource department. The clinicians who met the inclusion criteria were issued with an informed consent form. After signing and return of the consent form, the first author administered the study questionnaires for self-completion by each respondent.

### Data analysis

Data were entered into a Microsoft Excel spreadsheet using predetermined data variables, which included age, gender, cadre, and years of practice, level of knowledge of chronic pain, professional practice and attitudes amongst others. Data were cleaned by cross-checking the entries for each variable in the Excel spreadsheet against our study questionnaires. The clean data Excel spreadsheet was transferred to SPSS software for processing and statistical analysis. Descriptive statistics in terms of means and standard deviation (SD) were calculated and presented in the form of summary tables and charts.

### Ethical considerations

Ethical clearance was obtained from the National Commission for Science Technology and Innovation, Jomo Kenyatta University of Agriculture and Technology and Tenwek Hospital Ethical Review Committee (ERC). Participants were informed of their right to decline to participate and the process was purely voluntary. Confidentiality was maintained throughout the study and subsequent presentations. Consent from all potential participants was sought before questionnaires were administered. There were no names or identifiers linking questionnaires to respondent.

## Results

A total of 153 clinicians took part in our study of whom 79 (52%;) were male and 74 (48%) female. The majority 84 (55%) were younger than 30 followed by 47 (30%) who were aged between 31 and 40. Almost three quarters, 108 (71%) of the participants had less than 5 years of clinical experience. Only 27 (17%) had 6 years or more. The majority of participants 106 (70%) had attained a college diploma, half 77 (50%) of them were nurses and 23 (15%) were physicians’ assistants as illustrated in Table 2.

**TABLE 2:** Socio-demographic characteristics of participants (*n* = 153).

Characteristic	Frequency (*n*)	Percentage (%)
**Age group**		
< 30 years	84	55
31–40 years	47	30
41–50 years	19	13
> 50 years	3	2
**Years of clinical practice**		
1–5 years	108	71
6–10 years	27	17
> 11 years	18	12
**Level of education**		
College diploma	106	70
Bachelor’s degree	31	20
Master’s degree	8	5
Post graduate degree	8	5
**Clinician cadre**		
Nurses	77	50
Physicians’ assistants	23	15
Medical officers, residents and specialists	34	21
Physiotherapist	3	2
Anaesthetist	8	5
Dentist	3	2
Oncology team	5	3

### Level of knowledge and attitudes on assessment and management of chronic pain

The majority 139 (91%) of the participants had inadequate knowledge and attitudes (they scored below 75%) on assessment and management of chronic pain. Regarding their level of knowledge of the developmental changes in pain perception, few 48 (31%) participants were knowledgeable. Very few 16 (10%) participants had adequate knowledge and positive attitudes on the physiological basis of chronic pain management (see Table 3).

**TABLE 3:** Level of knowledge and attitudes on assessment and management of chronic pain (*n* = 153).

Variable	Adequate knowledge		Inadequate knowledge		Average score	
	*n*	%	*n*	%	SD	%
**Level of knowledge and attitudes on chronic pain**						
Physiological basis of pain	16	10	137	90	12.6	61.7
Psychological factors of pain perception	21	14	132	86	13.9	60.7
Developmental changes in pain perception	48	31	105	69	18.9	64.2
**Knowledge and attitudes on assessment and management**						
Assessment and measurement of pain	14	9	139	91	15.9	51.7
Cognitive/behavioural methods of pain relief	95	62	58	38	14.9	78.5
Pharmacological management of chronic pain	11	7	142	93	11.8	54.5
Overall scores	14	9	139	91	7.)	61.9

SD, standard deviation.

### Current practice of assessment and measurement, cognitive/behavioural and pharmacological intervention of chronic pain

**FIGURE 1:**
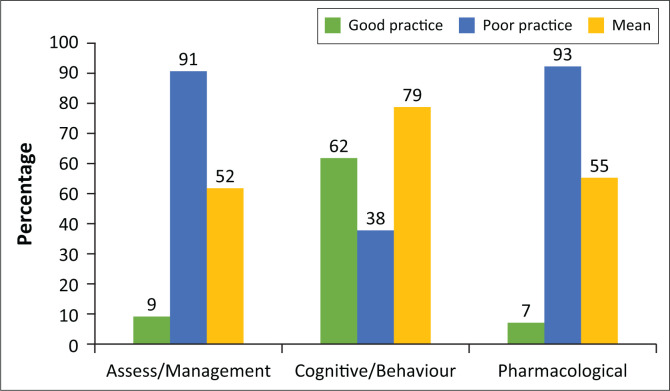
Current practice of assessment and measurement, cognitive/behavioural and pharmacological intervention of chronic pain (*n* = 153).

For cognitive/behavioural intervention of chronic pain, the majority of the participants (62% *n* = 95) used best practice, with a mean score of 79% (SD: 14.9%) whilst only 9% (*n* = 14) and 7% (*n* = 11) of the participants used best practice in assessing and pharmacologically managing patients with chronic pain, respectively (see Figure 1).

## Discussion

Our study is one of the few that has attempted to investigate the level of knowledge, attitudes and practices of clinicians managing chronic pain in a tertiary hospital in Kenya. Our results are a source of concern, as the majority of the participants had inadequate knowledge, negative attitudes and poor practices in managing chronic pain. These results are similar to studies that have also found clinicians who have inadequate knowledge, negative attitudes and poor practices regarding management of chronic pain (Al-Quliti & Alamri 2015; Clenzos et al. 2016; Kheshti et al. 2016). Our findings may have been influenced by variations in participants’ level and training programmes. As cited in other studies some clinicians may be inadequately prepared to assess and manage pain partly because of the form of training and clinical experiences (Kiwanuka & Masaba 2018; Nuseir et al. 2016; Yaqoob & Nasaif 2015). Furthermore, the majority of clinicians did not have much clinical experience (71%, *n =* 108), which is crucial to successfully manage chronic pain. This view is supported by Miró et al. (2019); Shipton et al. (2018); Enskär, Eaton and Harding (2007); Bouri et al. (2018) and Nuseir et al. (2016) who have shown that clinical experience and level of education influence clinician practice.

Although our study examined a wide range of clinical cadres, there was an uneven distribution amongst them; therefore, generalising our results to a larger population must be carried out cautiously because of the small study population and uneven distribution of clinical cadres. Despite this limitation, our results provide useful information on the selected care facility clinical cadre capacity for the assessment and management of clients with chronic pain and the need for the facility to generate an algorithm for chronic pain management to protect its clients’ rights to quality care.

Few studies in the region are similar to our findings. A Kenyan study by Jin (2015) (*n* = 96), which evaluated ‘the knowledge and attitudes of various healthcare workers regarding pain assessment and management in children’ in the country’s national referral hospital, found a significant knowledge gap amongst participants with over half (58.3%) performing poorly, whilst an Ugandan study by Kizza et al. (2016) found that 73.5% of clinicians were perceived to have inadequate knowledge in key concepts of pain management. Furthermore, a South African study by Clenzos et al. (2016) found only 14.5% of participants with adequate knowledge. The results of our study contrasts with a comparative study in the United Kingdom, South Africa and Sweden (*n* = 106) amongst clinicians working with children with cancer by Enskär et al. (2007) who found that participants had high levels of knowledge and appropriate attitudes towards pain management.

We found few participants used best practice in the assessment and measurement of pain, which is similar to Clenzos et al. (2016) and Al-Quliti and Alamri (2015) who found 4%; (*n* = 8) and 5.7% (*n* = 6) of participants, respectively, had adequate knowledge in this domain. Similarly, in the pharmacological domain, participants performed poorly, which corroborates the findings of Kheshti et al. (2016). Our results and similar results in other studies are disturbing because they point to a possibility that some pain interventions may be inadequate, which may increase the risk of chronification (Ranger, Johnston & Anand 2007; Subhashini, Vatsa & Lodha 2009).

Interestingly, most participants had good knowledge in the cognitive/behavioural aspect of chronic pain intervention compared with all other domains. This is a non-pharmacological aspect of pain intervention that focuses mainly on the psychological, social and educational aspects. It has been shown to be effective when combined with other modalities (Louw et al. 2016; Turk, Swanson & Tunks 2008). Undoubtedly, our study indicates the need to improve clinician’s level of knowledge, attitudes and practices to reduce the burden associated with chronic pain. A similar view has been expressed by Goldberg & McGee 2011; Salazar et al. 2016; Sessle 2012; Stewart et al. 2003. Furthermore, educational intervention particularly continuous medical education and adopting chronic pain guidelines have been recommended to improve clinician’s knowledge, attitudes and practices (Gustafsson & Borglin 2013; Mcnamara, Harmon & Saunders 2012). Our results form a baseline for further research in a wider population and may inform future review of healthcare policy, training curricula and practices in healthcare in our environment.

### Strengths and limitations

Our study had a good response rate of 83.5% (*n*− = 153) but was limited by uneven distribution of participants in the different cadres, those with small population may not be sufficient to generalise the results to the larger population.

### Implications and recommendations

We recommend studies to be conducted in a wider population to allow generalisation of the results educating clinicians on the assessment and management of patients with chronic pain, may help in improving their practices. Furthermore, training curricula for all levels of clinicians should emphasise chronic pain management to equip them with appropriate knowledge and skills necessary to assess and manage chronic pain.

## Conclusion

Patients with chronic pain continue to suffer because of clinicians’ inadequate knowledge, unhelpful attitudes and generally poor practice. Educating clinicians on best practice about assessment and management of chronic pain and encouraging self-initiated continuing medical education is critical. Our results suggest improvements in education curricula and the quality of healthcare in the region.
